# Inhibition of TLR4 Signalling-Induced Inflammation Attenuates Secondary Injury after Diffuse Axonal Injury in Rats

**DOI:** 10.1155/2016/4706915

**Published:** 2016-07-13

**Authors:** Yonglin Zhao, Yahui Zhao, Ming Zhang, Junjie Zhao, Xudong Ma, Tingqin Huang, Honggang Pang, Jiaxi Li, Jinning Song

**Affiliations:** ^1^Department of Neurosurgery, The First Affiliated Hospital of Xi'an Jiaotong University, 277 West Yanta Road, Xi'an, Shaanxi 710061, China; ^2^Department of Neurosurgery, The Second Affiliated Hospital of Xi'an Jiaotong University, No. 157 of Xiwu Road, Xi'an, Shaanxi 710004, China

## Abstract

Increasing evidence suggests that secondary injury after diffuse axonal injury (DAI) damages more axons than the initial insult, but the underlying mechanisms of this phenomenon are not fully understood. Recent studies show that toll-like receptor 4 (TLR4) plays a critical role in promoting adaptive immune responses and have been shown to be associated with brain damage. The purpose of this study was to investigate the role of the TLR4 signalling pathway in secondary axonal injury in the cortices of DAI rats. TLR4 was mainly localized in microglial cells and neurons, and the levels of TLR4 downstream signalling molecules, including TLR4, myeloid differentiation primary response gene 88, toll/IR-1-(TIR-) domain-containing adaptor protein inducing interferon-beta, interferon regulatory factor 3, interferon *β*, nuclear factor *κ*B (NF-*κ*B) p65, and phospho-NF-*κ*B p65, significantly increased and peaked at 1 d after DAI. Inhibition of TLR4 by TAK-242 attenuated apoptosis, neuronal and axonal injury, and glial responses. The neuroprotective effects of TLR4 inhibition were associated with decreases in the levels of TLR4 downstream signalling molecules and inflammatory factors, including interleukin-1*β*, interleukin-6, and tumour necrosis factor-*α*. These results suggest that the TLR4 signalling pathway plays an important role in secondary injury and may be an important therapeutic target following DAI.

## 1. Introduction

Diffuse axonal injury (DAI), also known as axonal damage or dysfunction, is regarded as the most common and important pathology of traumatic brain injury (TBI) [1]. Previous studies have ascribed typical axonal injury to shear stresses induced by rotational head acceleration/deceleration [2]. However, recent studies have shown that immediate mechanical tearing of axons is insufficient to cause the bulk of axonal injury [3–5]. More likely, the primary trauma evokes a cascade of changes that are detrimental to axons and may ultimately result in secondary axonal injury. Such pathological changes include mitochondrial alterations, ionic imbalances, demyelination, oxidative stresses, and lipid peroxidation [6]. Increasing evidence suggests that neuroinflammation and microglial activation may also contribute to cellular damage [7].

The toll-like receptors (TLRs) are pattern recognition receptors that belong to the interleukin receptor superfamily [8]. In the brain, TLRs are mainly localized in glial cells, such as microglia, astrocytes, and oligodendrocytes, and can produce proinflammatory cytokines when bound to their corresponding ligands [9]. Therefore, TLRs play a crucial role in regulation of the inflammatory response. Recently, TLRs have also been found to be expressed in neurons [10]. Of the TLR family members, TLR4 is the most important inflammation inducer. TLR4 recognizes both exogenous (pathogen-associated molecular pattern molecules) and endogenous ligands (damage-associated molecular pattern molecules, DAMP) and activates downstream adaptor signalling molecules, including myeloid differentiation primary response gene 88 (MyD88), toll/IR-1-(TIR-) domain-containing adaptor protein inducing interferon-beta (TRIF), interferon regulatory factor 3 (IRF3), interferon *β* (IFN-*β*), and nuclear factor *κ*B (NF-*κ*B), which eventually leads to production of a large number of inflammatory factors [11]. TLR4-mediated inflammatory reactions are associated with several central nervous system (CNS) disorders, such as cerebral ischaemia-reperfusion injury, Alzheimer disease, TBI, and intracerebral haemorrhage [12–16]. However, whether TLR4 participates in secondary injury following DAI remains unknown.

In the present study, we investigated the dynamic expression and localization of TLR4 and related proteins after DAI. We also assessed the effects of TLR-4 inhibition on apoptosis and other pathological changes in the cortex after injury. Our results showed that TLR4 was mainly localized in microglial cells and neurons and that its expression peaked at 1 d after DAI. Moreover, inhibition of TLR4 significantly attenuated apoptosis, neuronal and axonal injury, and glial responses. The effects of TLR4 inhibition were associated with decreases in the expression of signalling molecules downstream of TLR4, suggesting that TLR4 may be a potential target in DAI treatment.

## 2. Materials and Methods

### 2.1. Ethics

All experiments were conducted in accordance with the Guidelines and Suggestions for the Care and Use of Laboratory Animals formulated by the Ministry of Science and Technology of PRC and the Guidelines for the Care and Use of Laboratory Animals from the National Institutes of Health (NIH Publication number 80-23). This research was approved by the Biomedical Ethics Committee of Animal Experiments of Shaanxi Province in China. All injuries were inflicted under sodium pentobarbital anaesthesia, and all efforts were made to minimize suffering.

### 2.2. Animal Care and Grouping

Experiments were performed on male adult Sprague-Dawley (SD) rats (weighing 250–300 g, 8–10 weeks old), which were provided by the Experimental Animal Center of Xi'an Jiaotong University (License number SCXK (Shaanxi) 2006-001). The animals were housed in groups of 5 per cage in a room with a constant temperature of 22 ± 1°C, under a 12 hour light/dark cycle (lights on at 7:00 a.m.). Food and water were available ad libitum.

The rats were randomly divided into six groups as follows: (a) 12 rats were placed in the control group; (b) 6 rats were placed in the DAI 12 h group; (c) 12 rats were placed in the DAI 1 d group; (d) 12 rats were placed in the DAI 1 d+vehicle group, which was treated the same as the DAI 1 d group but received vehicle (i.v. in the tail vein, within 5 mins after DAI); (e) 24 rats were placed in the DAI 1 d+TAK-242 (0.1/0.5/1 mg/kg) groups, which were treated the same as the DAI 1 d+vehicle group but were administered different doses of TAK-242; and (f) 6 rats were placed in the DAI 3 d group. TAK-242 (Invivogen, San Diego, CA, USA) was formulated with 1% dimethyl sulfoxide and diluted with double-distilled water to a final concentration of 1 mg/mL.

The rats were acclimated to the environment over 3 days before DAI. Anaesthesia was administered immediately prior to injury. The DAI rats were initially comatose and regained consciousness after approximately half an hour. During the recovery period from anaesthesia, the rats were placed on a heating pad to maintain their body temperatures at 37°C to prevent hypothermia. All rats were pair-housed under temperature-controlled conditions, under 12 hour light/dark cycles, and had access to standard laboratory rodent chow and water ad libitum until euthanasia (at 12 h, 1 d, and 3 d after injury or drug treatment). For morphology staining, the rats were euthanatized with an intraperitoneal injection of 5% pentobarbital sodium (150 mg/kg) and perfused with 200 mL of normal saline, followed by 200 mL of 4% buffered paraformaldehyde (pH 7.4). The brain was removed, postfixed, embedded in paraffin, and cut into sections (5 *μ*m) with a rotary microtome. For western blotting and ELISA, the rats were perfused only with 200 mL of normal saline. After brain removal, cortical tissues were isolated and preserved in liquid nitrogen. In this study, 4 rats died within five minutes after DAI, likely as a result of traumatic cardiopulmonary arrest induced by severe damage to the brainstem. These rats were excluded and later replaced by new rats.

### 2.3. Animal DAI Model

A DAI model was established using a lateral head-rotation device [17]. After administration of anaesthesia with an intraperitoneal injection of 1% pentobarbital sodium (35 mg/kg), the rat head was horizontally secured to the lateral head-rotation device by two lateral ear bars, a head clip, and an anterior teeth hole, with the body positioned at a 30° oblique angle relative to the top of the laboratory table. When the trigger was pushed, the rat head was rapidly rotated 90°, resulting in sudden acceleration and deceleration. The control rats underwent only anaesthesia and fixation to the device and were not subjected to injury.

### 2.4. Haematoxylin and Eosin (H&E) Staining

Tissue sections were stained with haematoxylin and eosin, followed by dehydration, hyalinization, and fixation, and observed with a light microscope (Olympus, Tokyo, Japan) at 40x magnification. Three sections per animal were processed for H&E staining.

### 2.5. Immunohistochemical Staining and Semiquantitative Analysis

Immunohistochemistry was performed as described in a previous protocol [17]. Sections were dewaxed, hydrated, and incubated in 3% H_2_O_2_ deionized water for 10 minutes to block endogenous peroxidase, followed by antigen retrieval. The sections were placed in 0.01 mol/L citrate buffer and heated in a microwave oven at 95°C for 30 minutes, cooled to room temperature for 40 minutes, and washed three times with phosphate buffer saline (PBS) for 5 minutes each. Nonspecific protein binding was blocked by 30 minutes of incubation in normal goat serum at room temperature, followed by incubation with primary antibodies, including rabbit monoclonal *β*-amyloid precursor protein (*β*-APP) (1 : 300, Abcam, Cambridge, UK), mouse monoclonal tau46 (1 : 300, Cell Signalling Technology, Danvers, MA, USA), mouse monoclonal neuron-specific nuclear protein (NeuN) (1 : 400, Millipore, Billerica, MA, USA), mouse monoclonal glial fibrillary acidic protein (GFAP) (1 : 400, Cell Signalling Technology, Danvers, MA, USA), and rabbit polyclonal Iba-1 (1 : 300, Wako, Tokyo, Japan), at 4°C overnight. The sections were then incubated with goat anti-rabbit or mouse IgG-biotin (1 : 300) for 30 minutes and streptavidin-horseradish peroxidase for 30 minutes at 37°C. Diaminobenzidine was used as the chromogen, and haematoxylin was used as the counterstain. Finally, the sections were dehydrated in ethanol, cleared in xylene, and covered with coverslips. The results were observed under a microscope at 40x magnification.

Immunoreactivity was scored based on the number of positive cells and staining intensity, using Image-Pro Plus 6.0 software (Media Cybernetics, Rockville, MD, USA). Immunohistochemical scores (IHS) were determined by multiplying the quantity and staining intensity scores as follows: (1) the quantity was rated on a scale of 0–4, with no staining, 0; 1–10% of cells stained, 1; 11–50%, 2; 51–80%, 3; and 81–100%, 4; (2) staining intensity was rated on a scale of 0–3, with 0 = negative, 1 = weak, 2 = moderate, and 3 = strong. Theoretically, the scores could range from 0 to 12. An IHS of 9–12 was considered strong immunoreactivity, 5–8 was considered moderate, 1–4 was considered weak, and 0 was considered negative [18].

### 2.6. Immunofluorescence Staining

The brain tissue processing methods were the same as those described above (immunohistochemical staining). Rat brain sections were incubated overnight at 4°C with rabbit polyclonal TLR4 (1 : 200, Bioss, Beijing, China), mouse monoclonal NeuN (1 : 200), mouse monoclonal GFAP (1 : 300), and goat polyclonal Iba-1 (1 : 200, Abcam, Cambridge, UK). After washing, the brain sections were incubated with anti-rabbit, anti-mouse, or anti-goat secondary antibodies labelled with Alexa-Fluor 488 and Alexa-Fluor 647 (1 : 300, Abcam, Cambridge, UK) for 1 hour at room temperature. Nuclei were stained with 4′,6-diamidino-2-phenylindole (DAPI, 1 *μ*g/mL) for ten minutes. Stained sections were covered with coverslips, and final images were acquired using a fluorescence microscope (Molecular Devices, Sunnyvale, CA, USA).

### 2.7. Terminal Deoxynucleotidyl Transferase-Mediated Digoxigenin-dUTP-Biotin Nick-End Labelling (TUNEL) Assay

A DeadEnd*™* Fluorometric TUNEL System (Promega, Madison, Wisconsin, USA) was used to confirm apoptosis in the rat cortex. TUNEL was performed according to the manufacturer's instructions. Briefly, the brain tissue processing methods were the same as those described above (Immunohistochemical staining). Tissue sections were fixed via immersion of the slides in 4% methanol-free formaldehyde solution in PBS for 15 minutes. This was repeated once for 5 minutes, for a total of two PBS washes. A total of 100 *μ*L of 20 *μ*g/mL proteinase K was added to each slide to cover the tissue sections, which were then incubated for 10 minutes at room temperature. Then, the nuclei were stained with DAPI for ten minutes. Finally, the sections were examined with a fluorescence microscope. Data are expressed as the ratio of TUNEL to total nuclei.

### 2.8. Western Blotting Analysis

The western blotting methods were the same as those described in a previous protocol [17]. The rat cortices were homogenized with lysis buffer and a protease inhibitor cocktail. Samples were incubated on ice for 30 minutes, and the supernatants were recovered by centrifugation at 12 000 rpm at 4°C for 20 minutes. Nuclear protein was extracted using a kit from the Beyotime Institute of Biotechnology, according to the manufacturer's instructions. NF-*κ*B p65 and phospho-NF-*κ*B p65 expression was detected in the nuclear extract. Protein concentrations were determined with a Bradford protein assay kit, according to the manufacturer's instructions. Samples were incubated with 5x LDS buffer at 100°C for 5 minutes. Equal amounts of total protein (40 *μ*g) were resolved on 10% or 12% sodium dodecyl sulphate polyacrylamide gels and then transferred to polyvinylidene difluoride membranes (Millipore, Billerica, MA, USA). Then, the membranes were blocked with 5% skim milk powder in TBST buffer (0.05 M Tris pH 7.4, 0.15 M NaCl, 0.1% Tween20) for 1 hour at room temperature and incubated overnight with the appropriate primary antibodies, including monoclonal *β*-APP (1 : 1000), mouse monoclonal tau46 (1 : 500), mouse monoclonal NeuN (1 : 1000), mouse monoclonal GFAP (1 : 1000) and goat polyclonal Iba-1 (1 : 500), mouse monoclonal *β*-actin antibody (1 : 1000, Cell Signalling Technology, Danvers, MA, USA), rabbit monoclonal MyD88 (1 : 500, Cell Signalling Technology, Danvers, MA, USA), rabbit polyclonal TRIF (1 : 500, Abcam, Cambridge, UK), rabbit polyclonal IRF3 (1 : 500, Abcam, Cambridge, UK), goat polyclonal IFN-*β* (1 : 500, Santa Cruz, Dallas, Texas, USA), rabbit monoclonal NF-*κ*B p65 (1 : 1000, Cell Signalling Technology, Danvers, MA, USA), and rabbit monoclonal phospho-NF-*κ*B p65 (Ser536, 1 : 500, Cell Signalling Technology, Danvers, MA, USA). Next, the membranes were incubated with the corresponding IgG-HRP secondary antibody (1 : 5000; Abcam, Cambridge, UK) for 1 hour at room temperature, followed by washes in TBST. The membranes were visualized using a ChemiDoc MP System (Bio-Rad protein assay, Bio-Rad, Segrate, Italy) with ECL substrate (Millipore, Billerica, MA, USA). Densitometric quantification of the bands was performed using Image J software (version 1.29x: NIH, Bethesda, MD, USA).

### 2.9. Enzyme-Linked Immunosorbent Assay (ELISA)

Cortical tissues were homogenized with ethylene-diamine tetraacetic acid-free complete protease inhibitor cocktail tablets using 50 *μ*L/10 mg tissue. The homogenates were centrifuged at 14 000 rpm for 15 minutes at 4°C. The total protein content of each sample was determined using a Bradford protein assay kit. Cortical tissue lysates were analysed to determine the concentrations of inflammatory factors, including tumour necrosis factor-*α* (TNF-*α*), interleukin-1*β* (IL-1*β*), and interleukin-6 (IL-6), using an ELISA kit (R&D Systems, Minneapolis, MN, USA), according to the manufacturer's instructions. Each sample was run in duplicate, and data (pg protein) were normalized to mg of total protein.

### 2.10. Statistical Analysis

SPSS 18.0 (SPSS, Chicago, IL, USA) was used for statistical analysis. All data are presented as the mean ± SD. We used one-way ANOVA to compare numerical data in more than 2 groups, followed by LSD (L) to conduct a post hoc test. A *p* value less than 0.05 was considered statistically significant.

## 3. Results

### 3.1. Histopathological Changes in DAI Model Rats

**Figure 1:**
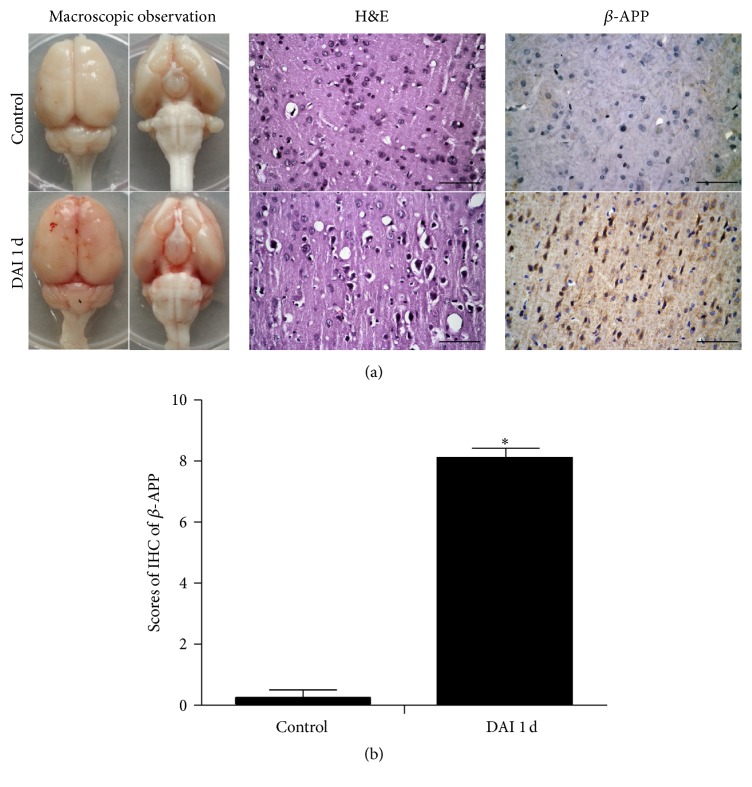
Pathological changes in the rat cortex after DAI. (a) Pathological changes after DAI were confirmed by macroscopic observations, H&E staining, and *β*-APP immunohistochemistry. Scale bar = 50 *μ*m. (b) The bar graphs show the *β*-APP expression results in the cortex (*n* = 6; ^*∗*^ *p* < 0.05, compared with control group). *β*-APP levels were assessed in 5 random fields (40x magnification) via immunohistochemical scores (IHS). Values are presented as the mean ± SD.

The brains of DAI rats showed varying degrees of subarachnoid haemorrhage without obvious cerebral contusion under macroscopic observation (Figure 1(a)). Neuronal pyknosis, swelling, torsion, cell body deformation, and extracellular space expansion were noted in the cortex at 1 d after DAI in H&E-stained sections (Figure 1(a)). No similar abnormal histopathological changes were observed in the control group. DAI was confirmed by *β*-APP immunohistochemistry staining, which is an excellent indicator of axonal injury. Increased numbers of *β*-APP-positive neurons were observed throughout the cortex at 1 d after DAI (Figures 1(a) and 1(b)).

### 3.2. Dynamic Expression of Proteins Related to the TLR4/NF-*κ*B Signalling Pathway after DAI

**Figure 2:**
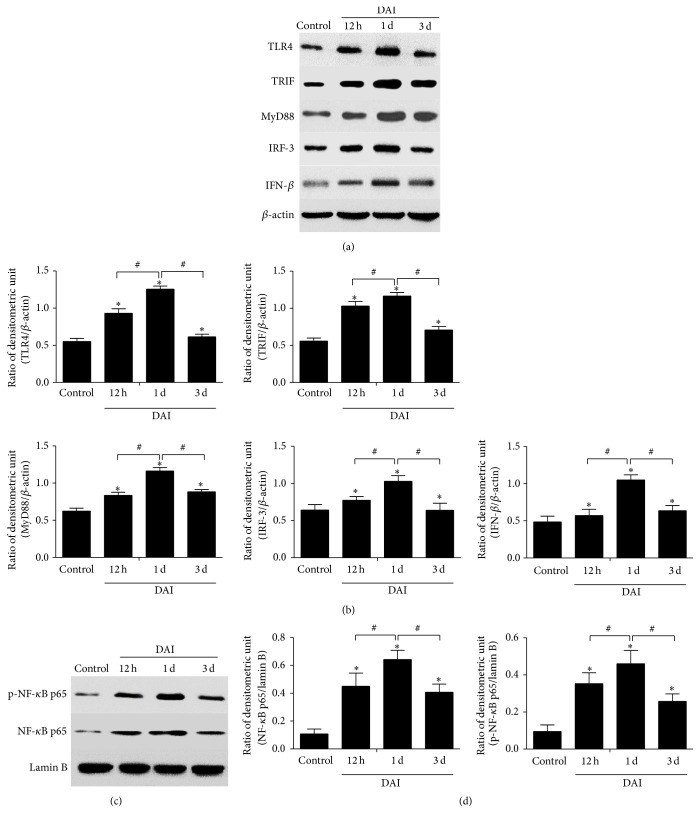
Dynamic expression of proteins related to the TLR4 signalling pathway after DAI. (a) Western blotting analysis was performed to measure the dynamic expression of TLR4, MyD88, TRIF, IRF3, and IFN-*β* at 12 h, 1 d, and 3 d after DAI. (b) The bar graphs show the cortical expression results for proteins related to the TLR4 signalling pathway. The expression of *β*-actin was used as an internal control. (c) Western blotting analysis was performed to measure the dynamic expression of NF-*κ*B and phospho-NF-*κ*B expression in nuclear extract at 12 h, 1 d, and 3 d after DAI. (d) The bar graphs show the results for NF-*κ*B and phospho-NF-*κ*B expression in the nuclear extract. The expression of lamin B was used as an internal control. Values are presented as the mean ± SD (*n* = 6; ^*∗*^ *p* < 0.05, compared with control group; ^#^ *p* < 0.05, compared with DAI 1 d group).

Western blotting analysis was performed to examine the expression of proteins related to the TLR4/NF-*κ*B signalling pathway, including TLR4, MyD88, TRIF, IRF3, IFN-*β*, intranuclear NF-*κ*B p65, and phospho-NF-*κ*B p65, at different time points. The results showed that compared to the control group, the expression of these proteins was significantly upregulated in the cortex at 12 h after DAI, peaked at 1 d, and then gradually decreased. However, until 3 d after DAI, the level of TLR4/NF-*κ*B pathway-related protein expression in the DAI group was still higher than that in the control group (Figure 2).

### 3.3. Localization of TLR4 at 1 d after DAI

**Figure 3:**
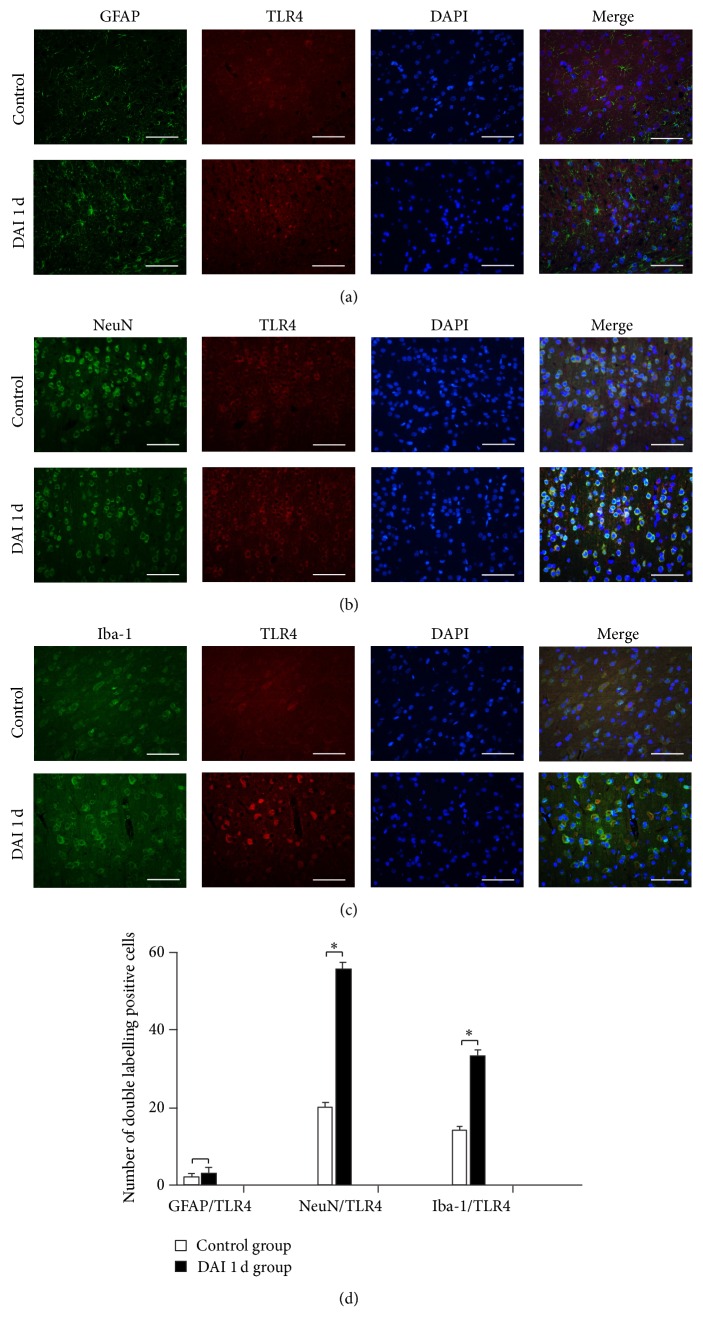
Localization of TLR4 in different cell types after DAI. (a–c) Double immunofluorescence staining was performed with an antibody to TLR2 and antibodies to GFAP (marker for astrocytes), NeuN (marker for neurons), and Iba-1 (marker for microglia). Antibody binding was demonstrated using the following fluorophore-conjugated secondary antibodies: TLR4 (red), NeuN (green), GFAP (green), and Iba1 (green). Nuclei were stained with DAPI (blue). Scale bar = 50 *μ*m. (d) The bar graphs show the results for the numbers of TLR4- and NeuN/GFAP/Iba-1-positive cells in the control and DAI 1 d groups. Values are presented as the mean ± SD (*n* = 3; ^*∗*^ *p* < 0.05).

To confirm the localization of TLR4, double-labelling experiments involving Iba1 (microglia), GFAP (astrocytes), NeuN (neuronal nuclei), and TLR4 were performed. TLR4- and GFAP-positive astrocytes were scarcely observed in the normal or DAI rat cortices (Figure 3(a)) via double-immunostaining. Surprisingly, the staining of NeuN and TLR4 via double-label immunofluorescence was more obvious in the DAI 1 d group than in the control group (Figure 3(b)), and TLR4- and Iba-1-positive cells were also more abundant in the DAI 1 d group than in the control group (Figure 3(c)). These findings suggest that the newly expressed TLR4 was mainly localized in microglial cells and neurons, instead of astrocytes. The expression of TLR4 was significantly increased in neurons and microglial cells in the rat cortex after DAI (Figure 3(d)).

### 3.4. Determination of the Optimal Dose of TAK-242

**Figure 4:**
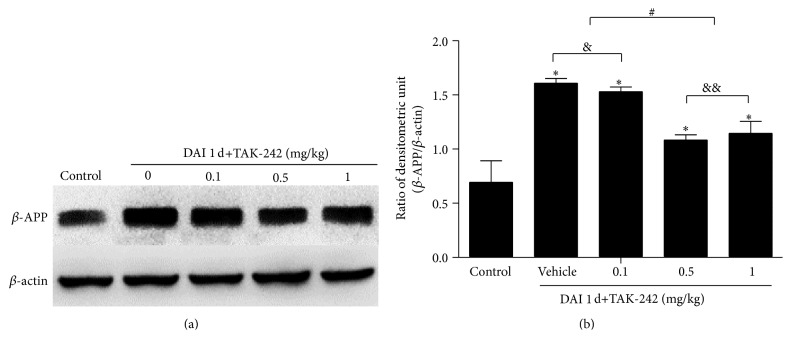
Determination of the optimal dose of TAK-242. (a) The effects of different doses of TAK-242 (0.1, 0.5, or 1 mg/kg) on DAI were determined via western blotting and measurement of *β*-APP expression. (b) The bar graphs show the *β*-APP expression results in the cortex after administration of different doses of TAK-242. The expression of *β*-actin was used as an internal control. Values are presented as the mean ± SD (*n* = 6; ^*∗*^ *p* < 0.05, compared with control group; ^&^ *p* > 0.05, compared with DAI 1 d+vehicle group; ^&&^ *p* > 0.05, ^#^ *p* < 0.05, compared with DAI 1 d+TAK-242 (0.5 mg/kg) group).

To determine its optimal dose, TAK-242 was intravenously injected into the caudal vein at doses of 0.1, 0.5, and 1 mg/kg immediately after DAI. The brain was harvested at 1 d posttreatment. The results showed that a dose of 0.5 mg/kg once daily achieved the maximum inhibitory effect on *β*-APP expression after DAI (Figure 4), which was consistent with previous in vivo studies reporting the anti-inflammatory/antioxidant and neuroprotective effects of TAK-242 in microglia exposed to hypoxia and in the rat frontal cortex after stress. Moreover, given that the expression of proteins related to the TLR4/NF-*κ*B signalling pathway peaked at 1 d after DAI, TAK-242 was administered only once at a dose of 0.5 mg/kg.

### 3.5. TLR4 Inhibition Significantly Ameliorated Apoptosis

**Figure 5:**
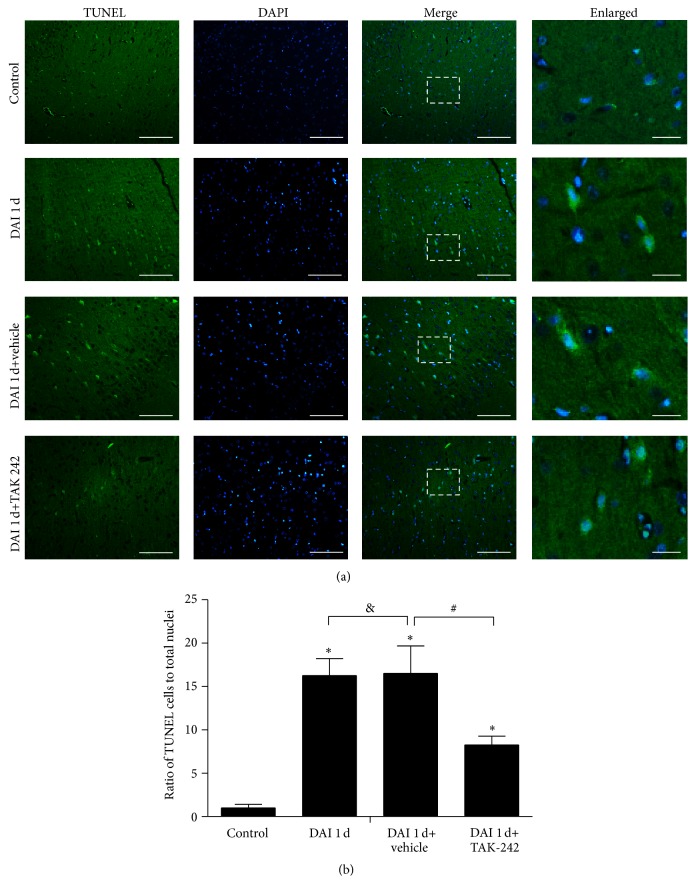
TLR4 inhibition significantly attenuated apoptosis after DAI. (a) TUNEL assay was used to detect apoptotic cells in the cortices of rats in the control, DAI 1 d, DAI 1 d+vehicle, and DAI 1 d+TAK-242 groups. TUNEL-positive cells were stained green, and the nuclei were stained with DAPI (blue). Scale bar = 100 *μ*m; enlarged figure scale bar = 20 *μ*m. (b) The bar graphs show the results regarding the numbers of apoptosis cells, which are expressed as the ratio of TUNEL to total nuclei. Positive cell levels were counted in 5 random cortical fields (40x magnification). Data are presented as the mean ± SD (*n* = 6; ^*∗*^ *p* < 0.05, compared with control group; ^#^ *p* < 0.05, ^&^ *p* > 0.05, compared with DAI 1 d+vehicle group).

TUNEL assay was performed to detect apoptosis in the cortices of rats. Limited numbers of TUNEL-positive cells were detected in the control group. TUNEL-positive cells were evident in the cortex in the DAI 1 d and DAI 1 d+vehicle groups, and no significant differences in TUNEL-positive cells were detected between these two groups. The number of apoptotic cells was significantly decreased in the DAI 1 d+TAK-242 group compared with the DAI 1 d group (Figure 5).

### 3.6. TLR4 Inhibitor Attenuated Secondary Damage after DAI

**Figure 6:**
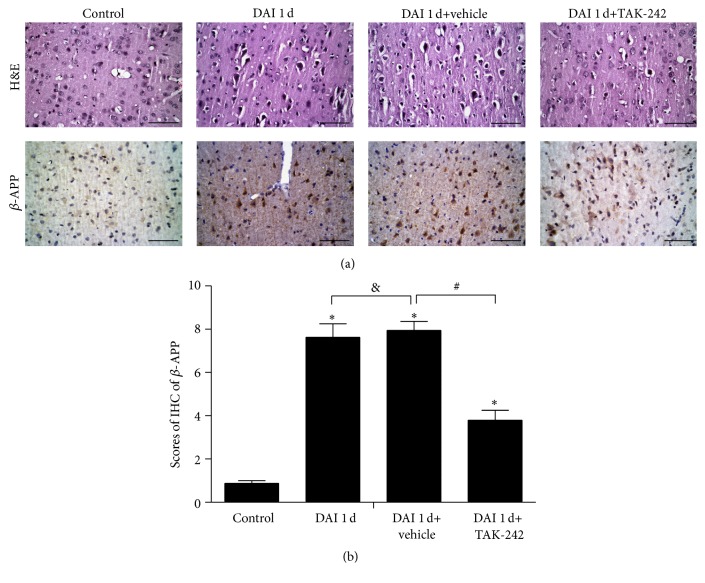
The protective role of TLR4 inhibition in DAI. (a) H&E staining and *β*-APP immunohistochemistry were performed to assess the pathological changes in the control, DAI 1 d, DAI 1 d+vehicle, and DAI 1 d+TAK-242 groups. (b) The bar graphs show the *β*-APP immunohistochemistry results for all groups. *β*-APP immunoreactivity was assessed in 5 random fields (40x magnification) via immunohistochemical scores (IHS). Data are presented as the mean ± SD (*n* = 6; ^*∗*^ *p* < 0.05, compared with control group; ^#^ *p* < 0.05, ^&^ *p* > 0.05, compared with DAI 1 d+vehicle group).

H&E staining showed that compared with the DAI 1 d and DAI 1 d+vehicle groups, neuronal pyknosis, swelling, torsion, cell body deformation, and extracellular space expansion were attenuated, and the numbers of *β*-APP-positive neurons and staining intensity were markedly decreased in the DAI 1 d+TAK-242 group. No significant differences in H&E staining or *β*-APP expression were observed between the DAI 1 d and DAI 1 d+vehicle groups (Figure 6).

**Figure 7:**
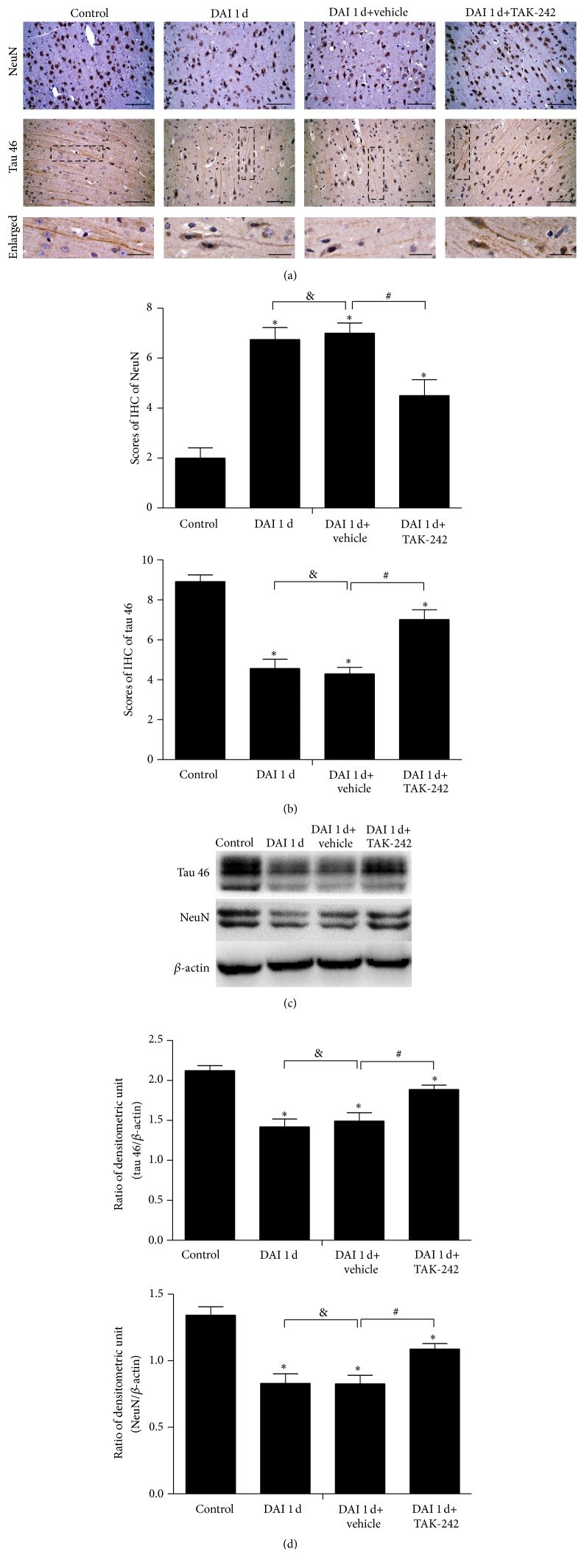
TLR4 inhibition attenuated neuronal and axonal injury. (a) Microtubule-associated protein tau immunoreactivity and neuronal nuclei protein (NeuN) immunoreactivity were assessed via immunohistochemistry in rat cortices in the control, DAI 1 d, DAI 1 d+vehicle, and DAI 1 d+TAK-242 groups. Scale bar = 50 *μ*m; enlarged figures scale bar = 20 *μ*m. (b) The bar graphs show the results for tau and NeuN immunoreactivity, as determined via immunohistochemistry. Tau and NeuN immunoreactivity was assessed in 5 random fields (40x magnification) via immunohistochemical scores (IHS). (c) The expression of tau and NeuN was measured via western blotting. (d) The bar graphs show the results for tau and NeuN expression, as assessed via western blotting. Data are presented as the mean ± SD (*n* = 6; ^*∗*^ *p* < 0.05, compared with control group; ^#^ *p* < 0.05, ^&^ *p* > 0.05, compared with DAI 1 d+vehicle group).

In the DAI and DAI+vehicle groups at 1 d after DAI, axons, which were virtually tau-negative, were found to display multiple regions of undulating distortions along their length. However, in the DAI+TAK242 1 d group, axons, whose shapes were similar to those of axons in the control groups, were tau-positive and showed fewer regions of undulating distortions (Figures 7(a) and 7(b)). This finding was further validated by western blotting (Figures 7(c) and 7(d)). In addition, NeuN expression, which was measured via western blotting and immunohistochemistry, was higher in the DAI 1 d+TAK-242 group than in the DAI 1 d and DAI 1 d+vehicle groups but was still lower than in the control group (Figure 7). No significant differences in tau and NeuN expression were observed between the DAI 1 d and DAI 1 d+vehicle groups (Figure 7).

### 3.7. TLR4 Inhibition Attenuated Glial Responses after DAI

**Figure 8:**
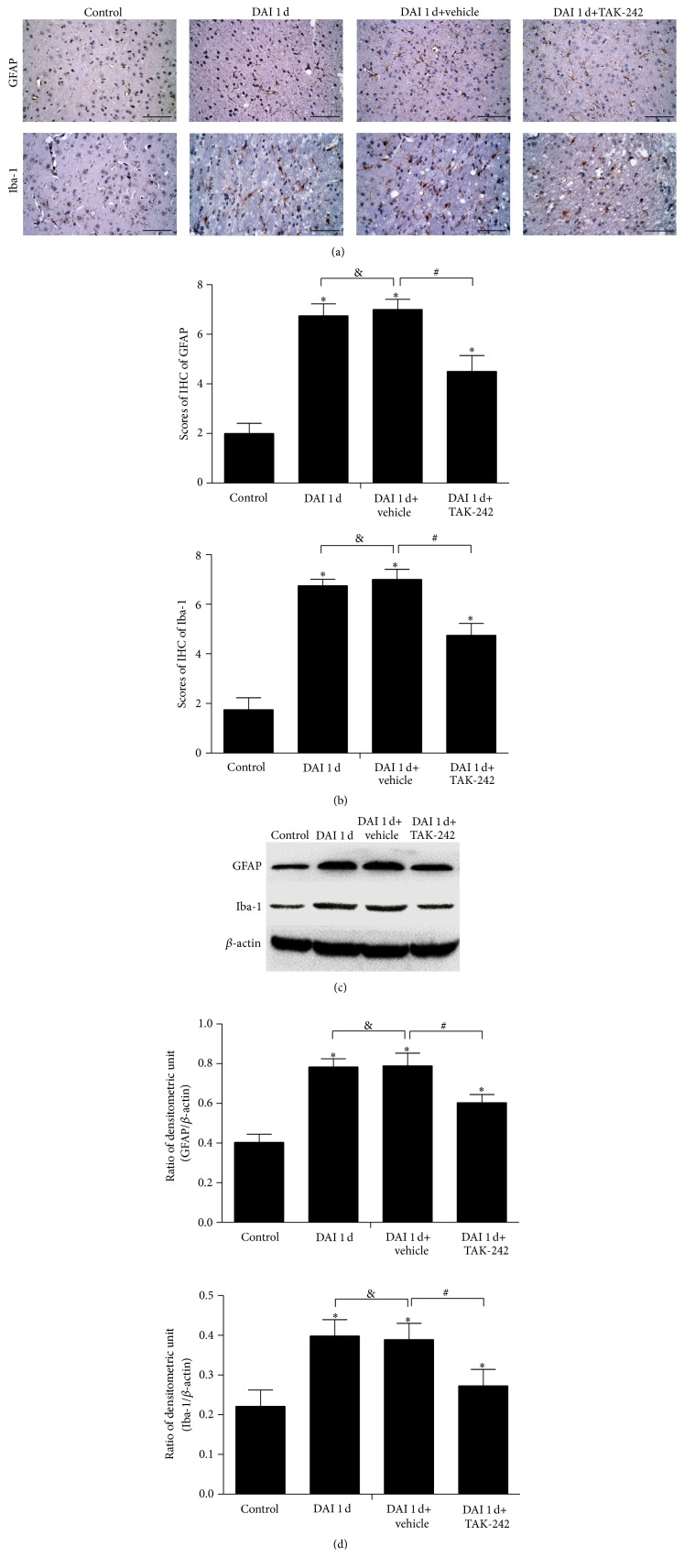
TLR4 inhibition attenuated glial responses. (a) The immunoreactivity of the astrocyte marker GFAP and that of the microglial cell marker Iba-1 were assessed via immunohistochemistry in rat cortices in the control, DAI 1 d, DAI 1 d+vehicle, and DAI 1 d+TAK-242 groups. Scale bar = 50 *μ*m. (b) The bar graphs show the results for GFAP and Iba-1 immunoreactivity, as determined via immunohistochemistry. GFAP and Iba-1 levels were assessed in 5 random fields (40x magnification) by immunohistochemical scores (IHS). (c) GFAP and Iba-1 expression was measured via western blotting. (d) The bar graphs show the results for GFAP and Iba-1 expression, as determined via western blotting. Data are presented as the mean ± SD (*n* = 6; ^*∗*^ *p* < 0.05, compared with control group; ^#^ *p* < 0.05, ^&^ *p* > 0.05, compared with DAI 1 d+vehicle group).

Compared with the control group, at 1 d after DAI, astrocyte and microglial cell staining was more robust, and the numbers of Iba-1-positive microglial cells and GFAP-positive astrocytes in the cortex were significantly increased. TAK-242 treatment significantly reduced the number of activated microglial cells and astrocytes at 1 d after DAI (Figures 8(a) and 8(b)). No significant differences in Iba-1 or GFAP expression were observed between the DAI 1 d and DAI 1 d+vehicle groups (Figures 8(a) and 8(b)). Similar results were confirmed by western blotting (Figures 8(c) and 8(d)).

### 3.8. TAK-242 Significantly Downregulated the Expression of Signalling Molecules Downstream of TLR4 and the Levels of Related Inflammatory Factors

**Figure 9:**
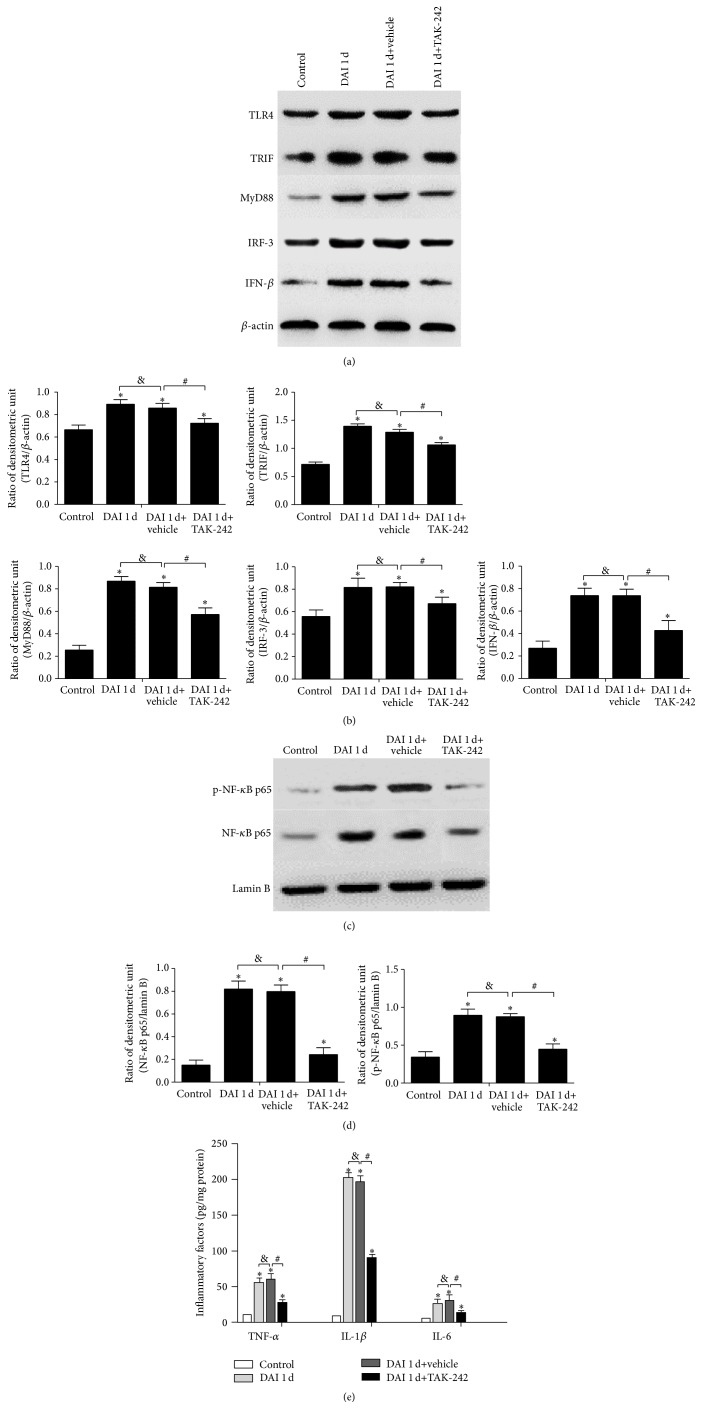
TAK-242 significantly downregulated the expression of signalling molecules downstream of TLR4 and reduced inflammatory factor levels. (a) The expression of several signalling molecules, including TLR4, MyD88, TRIF, IRF3, and IFN-*β*, in the control, DAI 1 d, DAI 1 d+vehicle, and DAI 1 d+TAK-242 groups, was measured via western blotting. (b) The bar graphs show the results for TLR4, MyD88, TRIF, IRF3, and IFN-*β* expression, as determined via western blotting. (c) Western blotting analysis was performed to examine the expression of NF-*κ*B and phospho-NF-*κ*B in nuclear extract from the control, DAI 1 d, DAI 1 d+vehicle, and DAI 1 d+TAK-242 groups. (d) The bar graphs show the results for NF-*κ*B and phospho-NF-*κ*B expression in the nuclear extract. The expression of lamin B was used as an internal control. (e) The levels of inflammatory factors, including TNF-*α*, IL-1*β*, and IL-6, were measured via ELISA in rat cortices in the control, DAI 1 d, DAI 1 d+vehicle, and DAI 1 d+TAK-242 groups. Data are presented as the mean ± SD (*n* = 6; ^*∗*^ *p* < 0.05, compared with control group; ^#^ *p* < 0.05, ^&^ *p* > 0.05, compared with DAI 1 d+vehicle group).

Western blotting analysis was performed to measure the expression of TLR4/NF-*κ*B pathway signalling molecules after DAI, including the levels of TLR4, MyD88, TRIF, NF-*κ*B, and phospho-NF-*κ*B after TAK-242 treatment. The results showed that TLR4 inhibition by TAK-242 significantly downregulated the expression of TLR4, MyD88, TRIF, IRF3, IFN-*β*, intranuclear NF-*κ*B p65, and phospho-NF-*κ*B p65 in the rat cortex compared with the DAI 1 d and DAI 1 d+vehicle groups, although their expression levels were still higher than those in the control group (Figures 9(a)–9(d)). No significant differences were observed between the DAI 1 d and DAI 1 d+vehicle groups.

Furthermore, the levels of inflammatory factors, such as TNF-*α*, IL-1*β*, and IL-6, in the cortices of rats after DAI were determined by ELISA. The results showed that the levels of these inflammatory factors were significantly increased in the DAI 1 d and DAI 1 d+vehicle groups compared with control group but were decreased in the DAI+TAK-242 group compared with the DAI 1 d+vehicle group (Figure 9(e)). No significant differences were observed between the DAI 1 d and DAI 1 d+vehicle groups.

## 4. Discussion

DAI is one of the leading causes of death and major morbidity following severe TBI. DAI not only results from physical injuries, as is often assumed, but also is also associated with pathological changes, such as perturbations in metabolism and electrochemistry and inflammation. Inflammatory responses have been shown to play an important role in neuronal injury after TBI [1]. Activation of TLR4 ultimately results in activation of NF-*κ*B, thereby inducing production of proinflammatory cytokines and promoting inflammation [11, 19]. In this study, the expression levels of proteins related to the TLR4/NF-*κ*B signalling pathway, including TLR4, MyD88, TRIF, IRF3, IFN-*β*, intranuclear NF-*κ*B p65, and phospho-NF-*κ*B p65, were all increased and peaked at 1 d after DAI, in accordance with a previous study [15]. TLR4 expression may peak at 1 d after DAI for several reasons. Our previous study indicated that cerebral microvasculature and axonal injury mainly occurred at 12–24 h after DAI and that these injuries may lead to the release of various types of DAMPs, such as high mobility group box 1 (HMGB1), heat shock proteins, *β*-amyloid, oxidized low-density lipoproteins, and others. HMGB1 protein levels were significantly decreased within the first 48 h after DAI compared with the control group, particularly within 24 h after injury, a finding indicative of the extracellular release of HMGB1 after DAI [20, 21]. All these results indicated that the TLR4 signalling pathway may be associated with secondary axonal injury.

It has been reported that TLR4 is expressed on CNS cells, including microglia, astrocytes, and endothelial cells [22]. Consistent with previous reports, in this study, TLR4 was also discovered on glial cells, primarily microglia. Controversy remains regarding whether TLR4 can be expressed on neurons under normal and pathological conditions. Our study showed that obvious localization of TLR4 on neurons was observed under both normal conditions and pathological conditions induced by DAI. Furthermore, TLR4 expression was significantly increased at 1 d after injury. Our results provide compelling evidence to support the idea that TLR4 can be expressed on both microglial cells and neurons under normal and pathological conditions.

TAK-242 inhibits TLR4 by binding to Cys747 in its intracellular domain [23]. Among the TLR4 antagonists that have been identified, TAK-242 crosses the blood-brain barrier most easily. In this study, we attempted to determine the effective dose of TAK-242 (0.1, 0.5, or 1 mg/kg), and the results showed that 0.5 mg/kg by intravenous injection once daily achieved the maximum inhibitory effect. This finding was also in accordance with that of a previous study [24]. The above dose has been used in a subsequent study by our group.

Histopathological identification of DAI depends upon visualization of abnormal axonal profiles. Historically, H&E staining has been successfully applied to identify damaged neurons and axons. In recent years, neuronal and axonal accumulation of *β*-APP has been recognized as a sensitive marker of trauma-induced axonal damage [25]. In this study, we performed H&E staining to identify pathologic changes, including neuronal pyknosis, swelling, torsion, cell body deformation, and extracellular space expansion, after DAI. *β*-APP expression was also significantly increased at 1 d after DAI compared to the control group. Inhibition of TLR4 significantly reduced these pathological changes, indicating that TLR4 inhibition exerts neuroprotective effects.

TLR4 inhibition has been shown to exert neuroprotective effects in many CNS diseases, including TBI [26–28]. Our results also revealed that blockade of the TLR4 signalling pathway attenuated axonal injury and apoptosis after DAI. NeuN and tau levels were significantly decreased in the cortex at 1 d after DAI, findings suggesting nuclear damage, microtubule disintegration, and axonal distortion [29]. Compared to the control group, the TLR4-inhibition group exhibited higher levels of NeuN and tau expression at the same time point, indicating that TAK-242 administration effectively stabilized microtubules and attenuated nuclear damage. Moreover, abnormal levels of Iba-1-positive microglial cells and GFAP-positive activated astrocytes, which are indictors of glial responses, were detected in the cortex after DAI. Administration of TAK-242 decreased Iba-1 and GFAP expression. These results demonstrated that TLR4 was directly responsible for neuronal death and the glial responses. Microglia can be activated by damage-associated molecules that recognize TLR4 and damage neighbouring cells through chronic overstimulation or prolonged inflammatory responses [30]. Microglial inflammation can be erroneous, amplified, and progressive. TAK-242 treatment may alleviate the damage to other CNS cells induced by overstimulated microglia, particularly neurons [31]. Furthermore, TLR4 was also observed in cortical neurons in the DAI groups. According to a previous study, necrotic neuron death can produce an immune response characterized by activation of TLR4-mediated pathways [32, 33]. Neuronal TLR4 expression may also predispose neurons to apoptosis under CNS pathological conditions. Therefore, we postulated that neuronal damage may be aggravated by increased neuronal TLR4 expression, in addition to the inflammation produced by microglial cells after DAI.

Activation of TLR4 signalling triggers two divergent inflammatory cascades, the MyD88 pathway, which leads to NF-*κ*B activation, and the TRIF pathway [22]. TLR4 is unique in that it can signal via MyD88-dependent or TRIF-dependent cascades. It has been suggested that to achieve a maximal inflammatory response, TLR4 may signal through both pathways [31]. In this study, the concentrations of several important inflammatory factors, such as those of IL-1 family members, IL-6 and TNF-*α*, increased after DAI, which implied that inflammatory responses are associated with DAI pathophysiology. Furthermore, the concentrations of these inflammatory factors were downregulated by inhibition of TLR4. Our results also demonstrated that TLR4 inhibition significantly reduced MyD88, TRIF, IRF3, and IFN-*β* expression and inhibited NF-*κ*B activation, thereby attenuating inflammatory factor expression and reducing neuronal apoptosis, neuronal damage, and glial responses, which may have ultimately improved DAI outcomes. Therefore, the damage induced by activation of TLR4 after DAI was mediated by upregulation of TLR4 downstream signalling molecules, resulting in secondary axonal injury after DAI.

## 5. Conclusion

In conclusion, our findings indicate that TLR4 inhibition with TAK-242 at 1 d after DAI successfully inhibited TLR4 signalling and effectively alleviated pathological changes, including apoptosis, neuronal and axonal injury, and glial responses, in a rat model of DAI. Our findings provide evidence that TLR4 signalling pathways exert remarkable cerebral deleterious effects after DAI and suggest that TLR4 inhibition holds great promise with respect to new DAI treatments.

## Supplementary Material

SD rats were randomly divided into control, DAI 12 h, DAI 1 d and DAI 3 d groups according to different time points after DAI. The expression of proteins related to the TLR4/NF-*β*B signalling pathway was detected in these groups and peaked at 1d post DAI. Then, TLR4 was found localized in neuron and microglia in DAI 1 d group. TLR4 inhibitor, TAK-242 was intravenously injected to DAI 1 d group at doses of 0.5 mg/kg next. Compared to DAI 1 d and DAI 1 d+vehicle groups, DAI 1 d+TAK-242 group showed decreased cell apoptosis, pathological changes, neuronal and axonal injury and glial reaction. Finally, the TLR4/NF-*β*B signalling pathway was involved in the neuroprotective effect induced by TAK-242.
