# Supplementary material for: Inhibition of TLR4 Signalling-Induced Inflammation Attenuates Secondary Injury after Diffuse Axonal Injury in Rats
Source: Mediators Inflamm. 2016 Jul 13;2016:4706915. doi: 10.1155/2016/4706915 (PMC4961816; doi:10.1155/2016/4706915)
Supplement: Supplementary file 1 — SD rats were randomly divided into control, DAI 12 h, DAI 1 d and DAI 3 d groups according to different time points after DAI. The expression of proteins related to the TLR4/NF-βB signalling pathway was detected in these groups and peaked at 1d post DAI. Then, TLR4 was found localized in neuron and microglia in DAI 1 d group. TLR4 inhibitor, TAK-242 was intravenously injected to DAI 1 d group at doses of 0.5 mg/kg next. Compared to DAI 1 d and DAI 1 d+vehicle groups, DAI 1 d+TAK-242 group showed decreased cell apoptosis, pathological changes, neuronal and axonal injury and glial reaction. Finally, the TLR4/NF-βB signalling pathway was involved in the neuroprotective effect induced by TAK-242. [file 4706915.f1.pptx]

## Slide 1
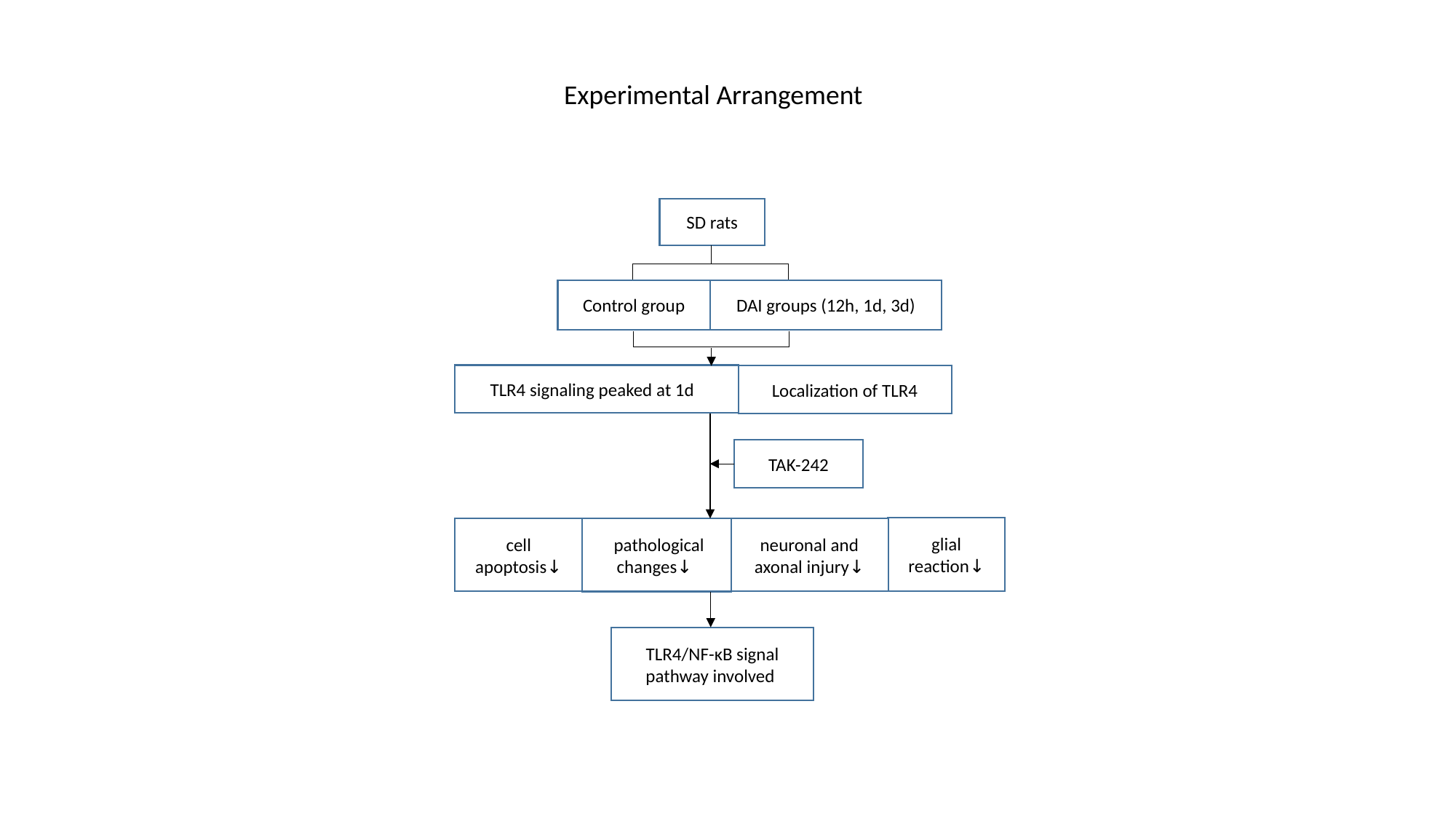

Experimental Arrangement
SD rats
DAI groups (12h, 1d, 3d)
Control group
TLR4 signaling peaked at 1d
Localization of TLR4
TAK-242
glial reaction↓
neuronal and axonal injury↓
cell apoptosis↓
 pathological changes↓
TLR4/NF-κB signal pathway involved
